# Aged Gut Microbiota Contributes to Systemical Inflammaging after Transfer to Germ-Free Mice

**DOI:** 10.3389/fimmu.2017.01385

**Published:** 2017-11-02

**Authors:** Floris Fransen, Adriaan A. van Beek, Theo Borghuis, Sahar El Aidy, Floor Hugenholtz, Christa van der Gaast – de Jongh, Huub F. J. Savelkoul, Marien I. De Jonge, Mark V. Boekschoten, Hauke Smidt, Marijke M. Faas, Paul de Vos

**Affiliations:** ^1^Top Institute Food and Nutrition, Wageningen, Netherlands; ^2^Department of Pathology and Medical Biology, University Medical Center Groningen, University of Groningen, Groningen, Netherlands; ^3^Cell Biology and Immunology Group, Wageningen University, Wageningen, Netherlands; ^4^Microbial Physiology, Groningen Biomolecular Sciences and Biotechnology Institute (GBB), University of Groningen, Groningen, Netherlands; ^5^Laboratory of Microbiology, Wageningen University, Wageningen, Netherlands; ^6^Laboratory of Pediatric Infectious Diseases, Radboud University Medical Center, Nijmegen, Netherlands; ^7^Nutrition, Metabolism and Genomics Group, Division of Human Nutrition, Wageningen University, Wageningen, Netherlands; ^8^Department of Obstetrics and Gynaecology, University Medical Center Groningen, University of Groningen, Groningen, Netherlands

**Keywords:** gut microbiome, immune system, inflammaging, germ-free mice, aging

## Abstract

Advanced age is associated with chronic low-grade inflammation, which is usually referred to as inflammaging. Elderly are also known to have an altered gut microbiota composition. However, whether inflammaging is a cause or consequence of an altered gut microbiota composition is not clear. In this study, gut microbiota from young or old conventional mice was transferred to young germ-free (GF) mice. Four weeks after gut microbiota transfer immune cell populations in spleen, Peyer’s patches, and mesenteric lymph nodes from conventionalized GF mice were analyzed by flow cytometry. In addition, whole-genome gene expression in the ileum was analyzed by microarray. Gut microbiota composition of donor and recipient mice was analyzed with 16S rDNA sequencing. Here, we show by transferring aged microbiota to young GF mice that certain bacterial species within the aged microbiota promote inflammaging. This effect was associated with lower levels of *Akkermansia* and higher levels of TM7 bacteria and *Proteobacteria* in the aged microbiota after transfer. The aged microbiota promoted inflammation in the small intestine in the GF mice and enhanced leakage of inflammatory bacterial components into the circulation was observed. Moreover, the aged microbiota promoted increased T cell activation in the systemic compartment. In conclusion, these data indicate that the gut microbiota from old mice contributes to inflammaging after transfer to young GF mice.

## Introduction

The gut microbiota is a highly complex and diverse community of bacteria that closely interacts with the epithelium and underlying immune cells in the gut (1). The bacterial divisions that dominate the human gut microbiota are *Firmicutes, Bacteroidetes, Actinobacteria*, and *Proteobacteria* (2). The dominance of these bacterial divisions is evolutionary conserved and has been confirmed in different mammalian species (3, 4). In recent years, it has become clear that the gut microbiota has a major impact on the immune system, metabolism, and even behavior of the host (5). Moreover, an imbalance in gut microbiota composition (dysbiosis) has been associated with several immunological, metabolic, and mental disorders (6). However, for the majority of these diseases it remains unclear whether dysbiosis is a cause or consequence of the disease.

In adults, the gut microbial community remains relatively stable (7). However, a number of studies have shown that gut microbiota composition is different in the elderly. For example, it has been demonstrated that *Firmicutes* was dominant in the gut microbiota of young individuals, whereas *Bacteroidetes* was more prevalent in the gut microbiota in the elderly (8, 9). Others found a decrease in anaerobes such as *Bifidobacteria*, but an increase in *Enterobacteria*, such as Escherichia *coli* in the elderly (10, 11). In people above 100 years old, an increase in pathobionts was observed (12). Also bacteria with anti-inflammatory properties such as *Faecalibacterium prauznitzii* were decreased in older individuals (13).

Concomitantly with microbiota changes, immunity becomes impaired in elderly (14). Elderly are known to be more susceptible to infections and mount less effective immune responses after vaccination. Moreover, homeostasis between pro-inflammatory and regulatory responses is lost, which results in a state of low-grade chronic systemic inflammation (14). The age-related chronic inflammation, which is called inflammaging, likely contributes to the pathology of several diseases typically associated with aging such as dementia, stroke, and cardiovascular diseases. In addition, advanced age has been reported to increase intestinal permeability in rodents and non-human primates and may subsequently enhance translocation of luminal bacterial products and induce inflammation (15, 16).

Whether age-induced microbiota changes are associated with inflammaging is not entirely clear, but there are some indications that intestinal microbes are involved in this process (17). To address the influence of the aged gut microbiota on the immune system, we transferred the gut microbiota from young or old conventional mice to germ-free (GF) mice. We demonstrate that the aged microbiota induced higher frequencies of several T helper (Th) cell subsets, in particular in the spleen. Moreover, expression of several inflammatory markers was elevated in the ileum after transferring microbiota of aged mice. Presumably translocation of bacterial components occurred, since the serum after transfer of aged microbiota contained higher levels of immunostimulatory bacterial components. Finally, gut microbiota composition analysis revealed differences in abundance of bacterial species such as *Akkermansia*, TM7, and *Proteobacteria*, which are potentially involved in the increased inflammatory potential of the microbiota of aged mice.

## Materials and Methods

### Study Design

All animal experiments were approved by the local ethical committee of the University of Groningen (project number 6543) and adhered to FELASA guidelines. The objective of this study was to determine whether the gut microbiota from aged mice contributes to the aging of the immune system. To this end, gut microbiota from young or old conventional mice was transferred to young GF mice. Effects on the immune system were compared between young or old conventional mice, GF recipients of young or old microbiota, and GF mice that remained GF throughout the experiment. Each experimental group consisted of 10 mice per group, except the control group of GF mice, which consisted of 5 mice per group. After an acclimatization period of at least 4 weeks, feces were freshly collected from the conventional mice. Feces from the same group was pooled and mixed in PBS. Next, 200 µl of 100 mg/ml of this mixture were given by oral gavage to GF mice of 12–14 weeks old. After transfer, recipient mice were individually housed in IVC cages for another 4 weeks. Mice were sacrificed on 10 different days, each day one mouse per group.

### Mice

Young (7–10 weeks) or old (17 months) C57BL/6JRccHsd conventional female mice were purchased from a commercial supplier (Envigo, Horst, the Netherlands). Female GF mice of 12–14 weeks were obtained from a breeding colony at the animal facility of Radboud University Nijmegen Medical Centre (Nijmegen, the Netherlands). All animals were put on an autoclaved Rat/mouse maintenance V153X R/M-H diet (Ssniff, Soest, Germany) directly after weaning in the case of GF mice, or directly after arrival in the case of conventional mice. The mice were kept on this diet throughout the experiment. Conventional mice were housed in IVC cages and GF mice were housed in GF isolators.

### Organ and Tissue Collection

Mice were sacrificed at the following ages: young conventional mice 16–19 weeks, old conventional mice 19–20 months, GF recipient mice 16–18 weeks, GF mice 13–15 weeks. Mice were anesthetized with isoflurane, bled, and sacrificed by cervical dislocation. Serum was collected and stored at −80°C. Colon content and a piece of terminal ileum were snap frozen in liquid nitrogen and stored at −80°C. In addition, spleen, Peyer’s patches (PPs), and mesenteric lymph nodes (MLNs) were collected for FACS analysis.

### Flow Cytometry

Single cell suspensions were obtained from spleen, PPs, and MLNs. Cells were stained with Fixable Viability Dye eFluor 506 (eBioscience, Vienna, Austria) for exclusion of dead cells. A-specific binding to Fc receptors was blocked by incubating the cells with anti-CD16/32 (clone 93, Biolegend, Uithoorn, the Netherlands) for 15 min on ice. For extracellular staining, cells were incubated with the desired mixture of antibodies for 30 min on ice. After washing, cells were fixed with FACS lysing solution (BD Biosciences, Breda, the Netherlands). For intracellular staining, fixed cells were permeabilized with PERM (eBioscience, Vienna, Austria) and subsequently stained with the desired antibodies for 30 min on ice. For identification of the different Th cell subsets, cells were stained with antibodies against: CD3e (clone 17A2), CD4 (clone GK1.5), T-bet (clone 4B10), RORγt (clone B2D), Gata-3 (clone TWAJ), CD25 (clone PC61), and Foxp3 (clone FJK-16S). Appropriate isotype controls were used to determine specificity of the staining. Samples were acquired with the FACSVerse (BD Biosciences, Breda) and analyzed with FlowJo software (FlowJo, LLC, Oregon, USA).

### Transcriptome Microarray

A piece of terminal ileum from each mouse was snap frozen in liquid nitrogen and stored afterward at −80°C. From these samples, RNA was isolated with the RNeasy kit (Qiagen, Valencia, CA, USA) and whole-genome gene expression was analyzed with Affymetrix GeneChip Mouse Gene 1.1 ST arrays as described previously (18). The gene expression datasets were deposited in NCBI’s Gene Expression Omnibus (GEO) and are accessible through GEO Series accession number: GSE104063.

### HEK293 toll-like receptor (TLR)2/TLR4 Assay

Human Embryonic Kidney 293 cells stably transfected with mouse TLR2/CD14 or TLR4/MD-2/CD14 and the secreted embryonic alkaline phosphatase reporter coupled to the NF-kB/AP-1 promoter were purchased from Invivogen (San Diego, CA, USA). Every cell line was grown at 37°C, 5% CO_2_ in DMEM medium (Lonza B.V., Basel, Switzerland), supplemented with 4.5 g/l glucose, 10% heat-inactivated FBS, 2 mM l-glutamine, 50 U/ml penicillin, 50 mg/ml streptomycin, and 100 mg/ml Normocin. After two passages, the cells were cultured in the presence of HEK-Blue selection medium (Invivogen, San Diego, CA, USA) in order to maintain the transfected constructs. Cells were stimulated with 2.5% mouse serum for 20 h at 37°C, 5% CO_2_. As a control, cells were stimulated in triplo with medium only (negative control), 100 ng/ml lipopolysaccharide (LPS)-EK (InvivoGen; positive control for TLR4), or 10^7^ bacteria/well of heat-killed Listeria monocytogenes (InvivoGen; positive control for TLR2). Next, 20 µl medium from each well was aliquoted and mixed with 180 µl QUANTI-Blue reagent (Invivogen, San Diego, CA, USA). After incubation at 37°C for 2 h, OD at 650 nm was measured with a microplate absorbance spectrophotometer (Bio-Rad Laboratories, Veenendaal, the Netherlands).

### Microbiota Analysis

Fresh feces samples obtained just after defecation were collected from all mice at different time points during the experiment. In addition, colonic content samples from these mice were collected at the end of the experiment. All samples were snap frozen in liquid nitrogen and stored at −80°C. These samples were used for 16S rRNA gene analysis for microbiota profiling with barcoded amplicons from the V1–V2 region of 16S rRNA genes as described previously (18). Briefly, amplicon pools were 250 bp paired-end sequenced using Illumina Miseq (GATC-Biotech, Konstanz, Germany). The Illumina Miseq data analysis was carried out with a workflow employing the Quantitative Insights Into Microbial Ecology pipeline (19) and a set of in-house scripts as described before for Illumina Hiseq 16S rRNA gene sequences (Hermes et al., manuscript in preparation).

### Statistics

Flow cytometry data and HEK293 TLR assay data are expressed as means, error bars represent SEM. To verify whether data were normally distributed, the Kolmogorov–Smirnov test was performed. In cases where data were not normally distributed, data were log transformed before analysis. For comparing two groups, the unpaired two-tailed Student’s *t* test was used. For comparing more than two groups with each other, one way ANOVA was performed followed by the Bonferroni test to compare specific groups. *P*-values below 0.05 were considered significant. All tests were performed with Graphpad software (Prism, La Jolla, CA, USA).

## Results

### Microbiota of Old Mice Enhances CD4^+^ T Cell Differentiation in the Spleen

In order to investigate how aging influences the interplay between the gut microbiota and the immune system of the host, we transferred gut microbiota from young (11–14 weeks) or old (18 months) conventional mice to young GF mice (12–14 weeks). Four weeks later, the mice were sacrificed and the frequency of the different CD4^+^ Th subsets were identified in the PPs, MLNs, and the spleen by flow cytometry (see Figure S1 in Supplementary Material for gating strategy). Conventional mice, aged 11–14 weeks, or 18 months served as control.

**Figure 1:**
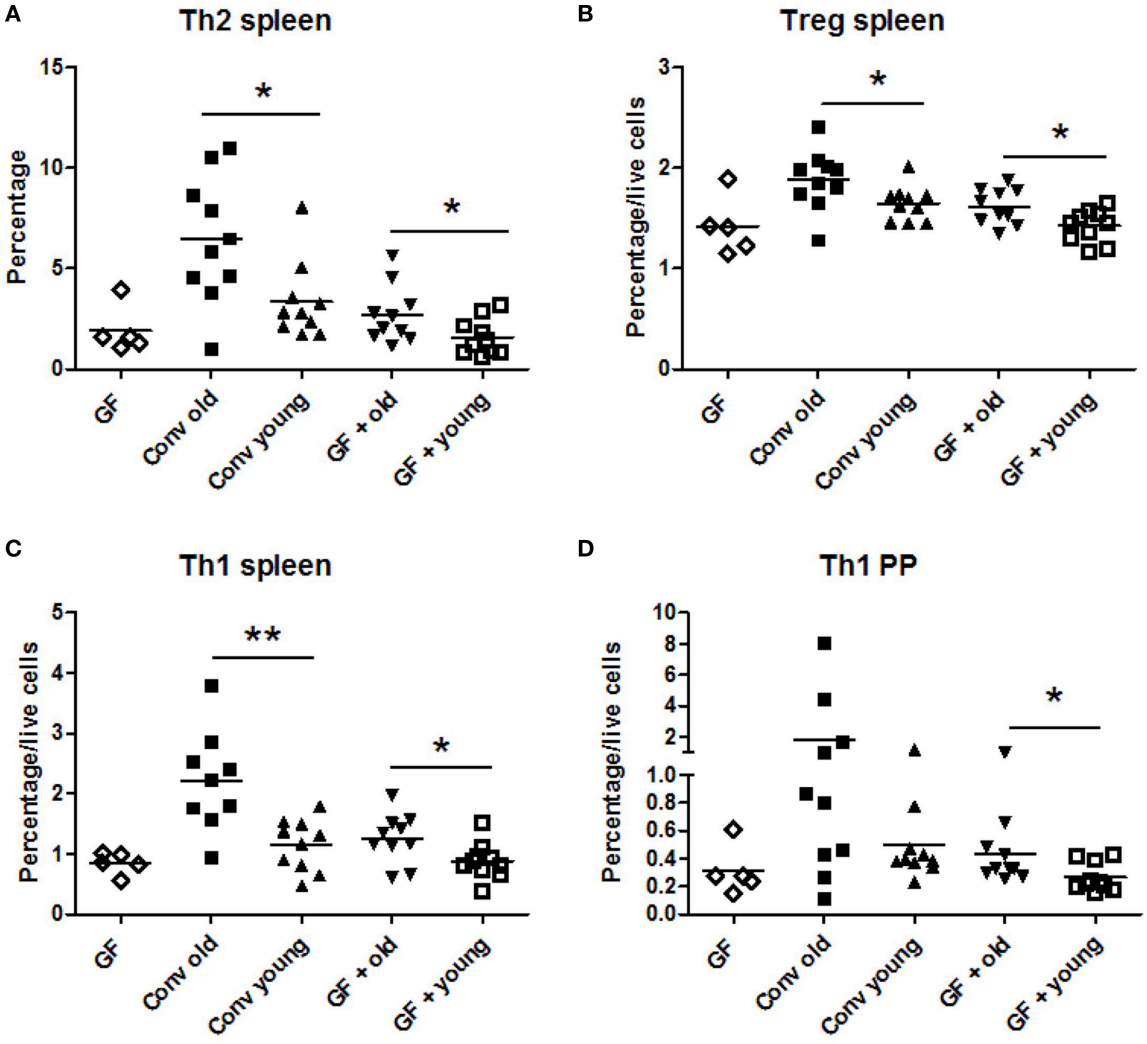
Old microbiota induces higher frequencies of T helper (Th) subsets in the spleen. Spleen, mesenteric lymph node, and Peyer’s patch (PP) CD4^+^ T cell populations were analyzed with flow cytometry after isolation from young or old conventional (conv) mice (*n* = 10), germ-free (GF) recipient mice of young or old microbiota (*n* = 10), and GF control mice (*n* = 5). **(A)** Percentage of splenic CD4^+^ T cells expressing GATA-3 (Th2). **(B)** Percentage among total live cells of splenic CD4^+^ T cells expressing CD25 and Foxp3 (T_reg_). **(C)** Percentage among total live cells of splenic CD4^+^ T cells expressing T-bet (Th1). **(D)** Percentage among total live PP cells of CD4^+^ T cells expressing T-bet (Th1). All data are expressed as means. *P < 0.05, **P < 0.01.

In conventional mice, a higher frequency of Th2 cells was found in the spleen of old mice compared with young mice (Figure 1A). This enhanced Th2 frequency could be induced by transfer of the old microbiota to young GF mice and was not observed when young microbiota was transferred. Also the high T_reg_ (Figure 1B) and Th1 (Figure 1C) numbers in spleens of old conventional mice could be induced by transfer of old microbiota. GF mice, which received the old microbiota had a higher frequency of splenic T_regs_ (Figure 1B) and Th1 cells (Figure 1C) than GF mice which received the young microbiota. No differences were observed in Th17 cells (data not shown). Furthermore, in the PPs and MLNs no differences were observed in Th frequencies (data not shown), except for Th1 cells in PPs, which were significantly higher in GF mice after transfer of the old microbiota (Figure 1D) when compared with GF mice after transfer of microbiota of young mice. In conclusion, the old microbiota enhanced CD4^+^ T cell differentiation or distribution of several Th subsets, in particular in the systemic compartment.

### Microbiota of Old Mice Upregulates Inflammation-Associated Immune Pathways in the Ileum

**Figure 2:**
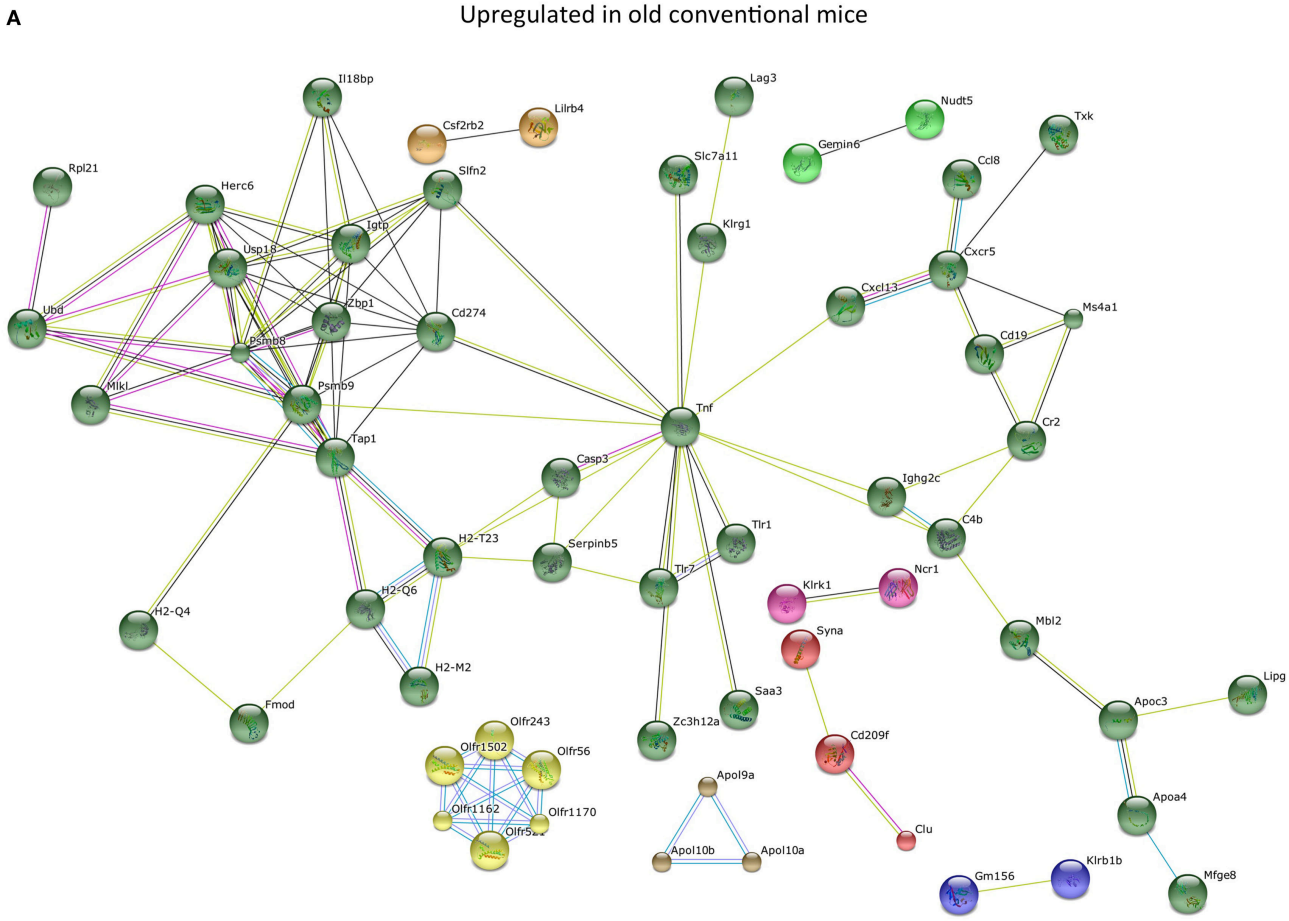

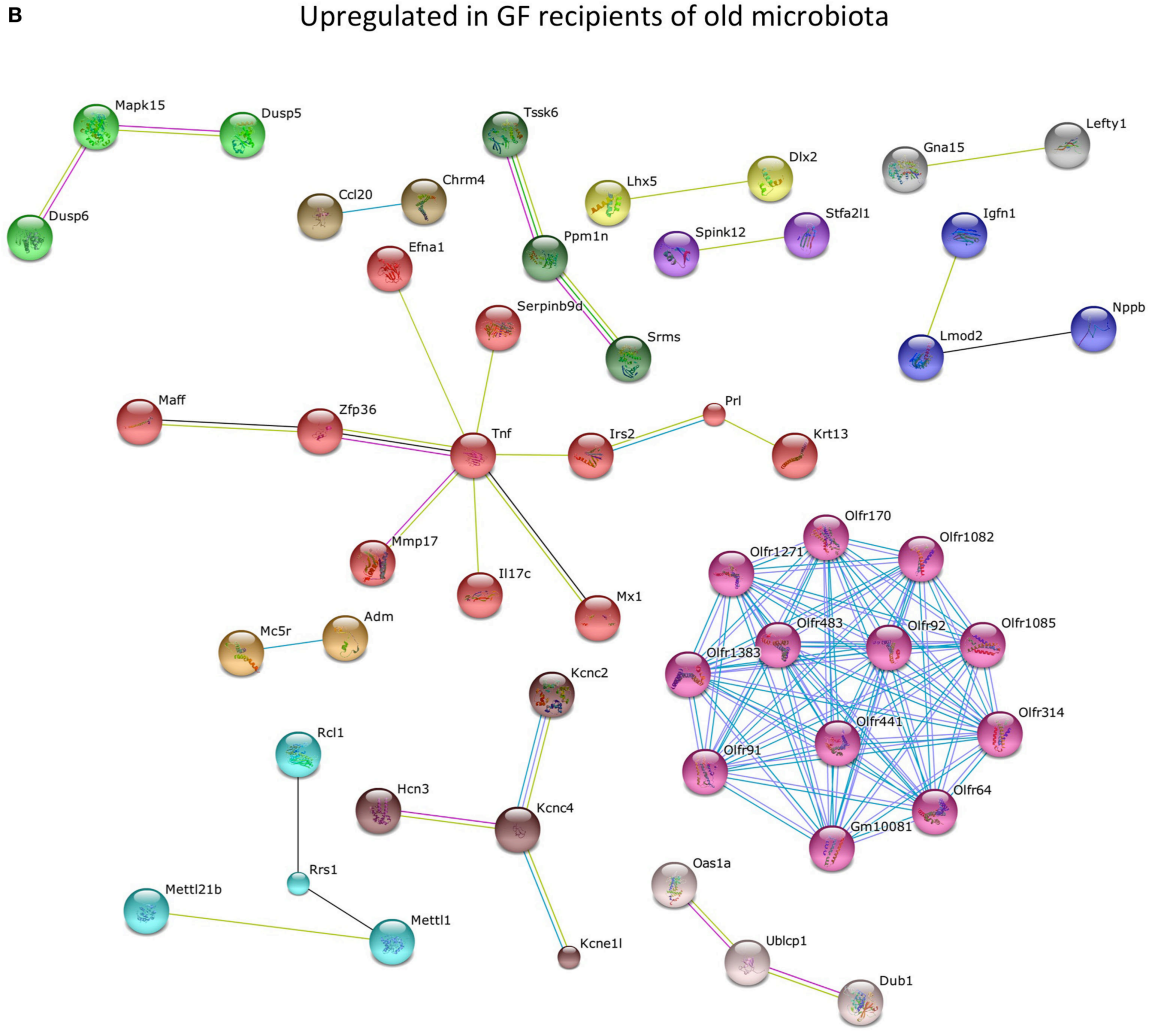
Old microbiota upregulates several immune pathways in the ileum. Whole-genome gene expression in the ileum of mice (*n* = 5 per group) was assessed with Affymetrix GeneChip Mouse Gene 1.1 ST arrays. The most highly upregulated genes were analyzed with the STRING database. Only genes with at least one interaction are shown. The following interactions are indicated: from curated databases (blue), experimentally determined (pink), textmining (yellow), co-expression (black), and protein homology (purple). **(A)** Genes that were upregulated (*P* < 0.05, fold-change >1.3) in the ileum of old conventional mice compared with young conventional mice. **(B)** Genes that were upregulated (*P* < 0.05, fold-change >1.2) in the ileum of germ-free (GF) mice that received old microbiota compared with GF mice that received the young microbiota.

To study the effect of the microbiota on the host in an unbiased manner, we performed genome-wide gene expression analysis of the ileum with microarray. Genes that were significantly higher expressed in the ileum of old conventional mice compared with young conventional mice were analyzed with the STRING database (20). We identified a large cluster of genes involved in the immune response that were upregulated in the ileum of old conventional mice (Figure 2A). The function of these genes included antigen processing and presentation, activation of the complement pathway, recognition of microbe-associated molecular patterns, and migration of B cells. TNF-α was in the center of this network, which might suggest that TNF-α plays an important role in these processes.

Also genes that were significantly higher expressed in GF mice that received the old microbiota compared with recipients of young microbiota were analyzed with the STRING database. Also here we identified a cluster of genes with TNF-α in the center of the network (Figure 2B). These results might suggest that TNF-α production is specifically enhanced by the old microbiota.

### Identification of Immune Pathways Specifically Affected by Old and Young Microbiota

**Figure 3:**
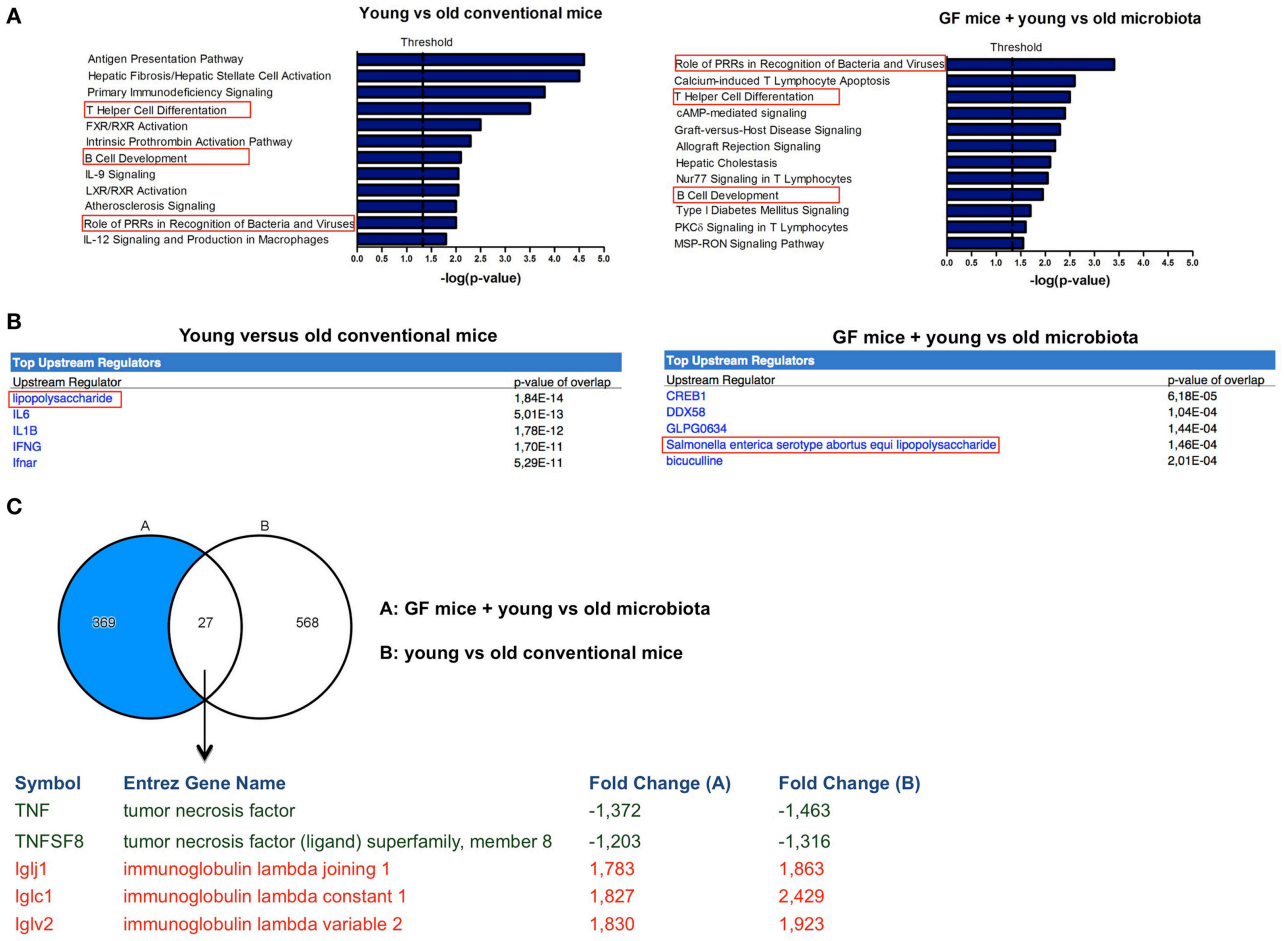
Markers of inflammation are upregulated in the presence of old microbiota. Whole-genome gene expression in the ileum of mice (*n* = 5 per group) was assessed with Affymetrix GeneChip Mouse Gene 1.1 ST arrays. Genes that were significantly differentially expressed (*P* < 0.05 and fold-change >1.2) between young and old conventional mice, or germ-free (GF) recipients of young or old microbiota were analyzed with Ingenuity Pathway Analysis. **(A)** Canonical pathways that were most significantly affected by age or after transfer of aged microbiota. **(B)** Most significantly predicted upstream regulators by comparing young and old conventional mice, or GF recipients of young or old microbiota. **(C)** Venn diagram of differentially expressed genes between young and old conventional mice, and GF recipients of young or old microbiota.

Microarray data were further analyzed with Ingenuity Pathway Analysis (IPA), only focusing on genes that were significantly differentially expressed (*P* < 0.05, fold-change >1.2 or <−1.2) when comparing old versus young conventional mice, or GF recipient mice that received young versus old microbiota. Interestingly, we observed three canonical pathways that were significantly affected both in the conventional mice and in the GF recipient mice (Figure 3A). The canonical pathways “role of PRRs in recognition of bacteria and viruses,” “Th cell differentiation,” and “B-cell development” were upregulated in old conventional mice compared with young conventional mice and also in GF recipients of old microbiota compared with recipients of young microbiota. Therefore, these pathways might be in particular influenced by the microbiota during aging.

Also predicted upstream regulators were identified with IPA. Upstream regulators are the upstream transcriptional regulators that potentially explain the observed gene expression differences in the dataset. The most significantly predicted upstream regulator that could cause the gene expression profile in old conventional mice in comparison with young conventional mice was LPS (Figure 3B). Importantly, LPS from *Salmonella enterica* was among the most significantly predicted upstream regulators of old microbiota after transfer to GF mice (Figure 3B). Thus, LPS is a component of the old microbiota that is possibly involved in mediating its effects on the immune system of the host.

To further identify the genes that were specifically influenced by the old microbiota, we compared the genes that were differentially expressed between young versus old conventional mice and GF recipients of young versus old microbiota (Figure 3C). We identified 27 genes that were differentially expressed in both datasets. This list of genes was further narrowed down to genes that were up- or down-regulated in both datasets and are known to play a role in the immune response. As mentioned above, TNF-α was upregulated both in old conventional mice and in GF recipients of old microbiota. Also, TNFSF8, which is the ligand for CD30, was more highly expressed in these groups of mice. On the other hand, several genes encoding for the lambda immunoglobulin light chain were more highly expressed both in young conventional mice and in GF recipients of young microbiota.

### Higher Amounts of Bacterial Components in Systemic Circulation after Old Microbiota Transfer

**Figure 4:**
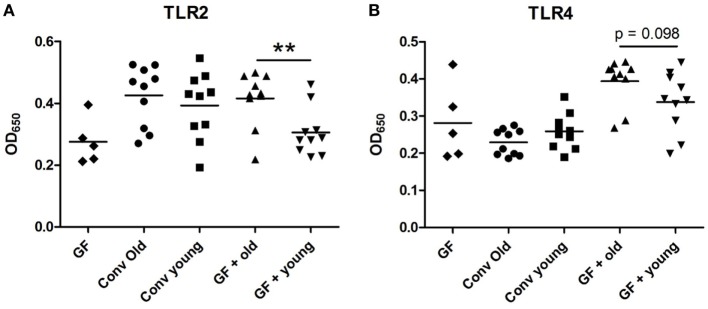
Transfer of old microbiota enhances inflammatory bacterial components in serum. Human Embryonic Kidney 293 cells transfected with mouse toll-like receptor (TLR)2/CD14 **(A)** or mouse TLR4/MD-2/CD14 **(B)** were stimulated with 2.5% serum from young or old conventional (conv) mice (*n* = 10), germ-free (GF) recipient mice of young or old microbiota (*n* = 10), and GF control mice (*n* = 5). Activation of these receptors was measured with a secreted embryonic alkaline phosphatase reporter coupled to the NF-kB/AP-1 promoter. All data are expressed as means. **P < 0.01.

As LPS was a prominent predicted upstream regulator in the ileal IPA analysis, we investigated whether there were innate immune activating components in sera of animals receiving old microbiota. These components can possibly be transferred from the intestine by translocation of bacterial components due to a compromised intestinal barrier (21, 22). To this end, we incubated the sera of these mice with HEK293 cells transfected with TLR 2 or TLR4. Activation of NFκB was measured with a reporter gene. No differences were observed between old conventional mice and young conventional mice. However, the sera from GF mice, which had received old microbiota showed significantly higher activation of TLR2 compared with sera from recipients of young microbiota (Figure 4A). A similar trend was observed for TLR4 activation, although this difference did not reach statistical significance (Figure 4B). In summary, these data indicate that old microbiota transfer leads to increased translocation of inflammatory bacterial products into the circulation.

### Bacterial Groups Associated with Increased Inflammatory Potential of Old Microbiota

**Figure 5:**
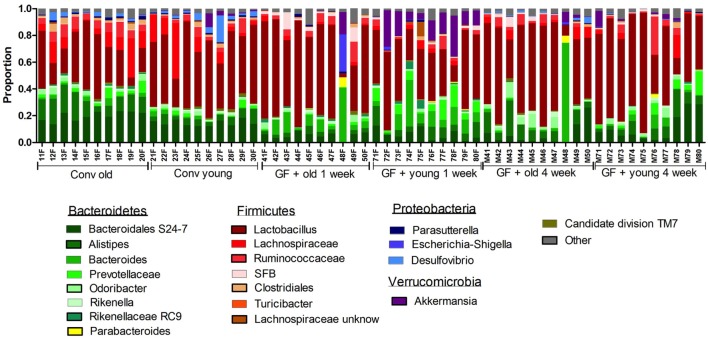
Gut microbiota composition in conventional and conventionalized mice. Fecal samples were collected from conventional (conv) mice (*n* = 10) at the time of transfer to the germ-free (GF) recipient mice, or from the GF recipient mice (*n* = 10) 1 and 4 weeks after the transfer. Gut microbiota composition was analyzed with 16S rDNA sequencing and data are presented as the relative abundance of the different bacterial groups for each individual mouse. The most highly abundant bacterial groups are indicated.

**Figure 6:**
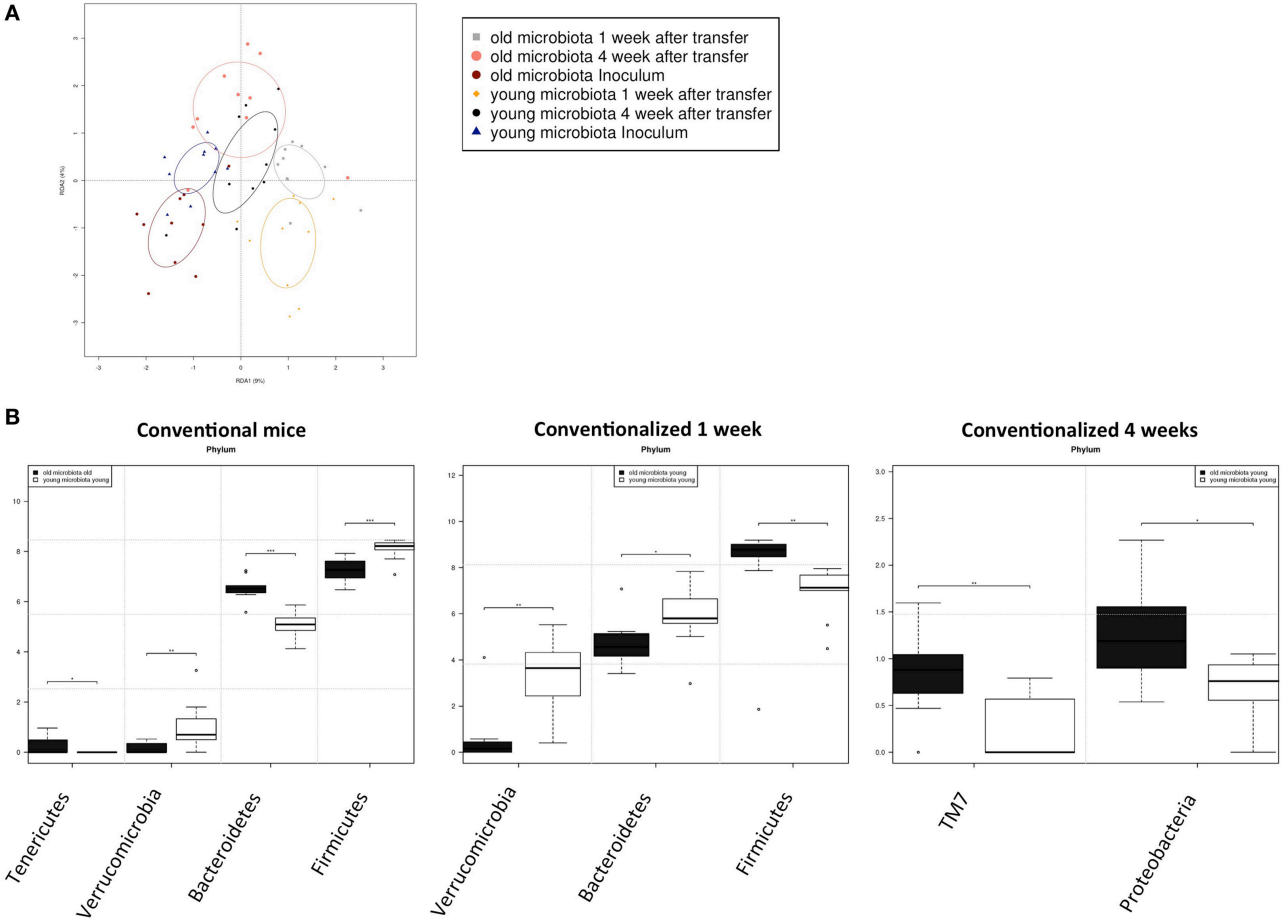
Transfer of aged microbiota to germ-free (GF) mice leads to altered gut microbiota composition. Fecal samples were collected from conventional (conv) mice (*n* = 10) at the time of transfer (inoculum) to the GF recipient mice, or from the GF recipient mice (*n* = 10) 1 and 4 weeks after the transfer. Gut microbiota composition was analyzed with 16S rDNA sequencing. **(A)** Redundancy analysis of gut microbiota composition of the different experimental groups at the genus level. **(B)** Bacterial phyla that were significantly different in abundance in young or old conventional mice, or in GF mice conventionalized with young or old microbiota after 1 or 4 weeks *P < 0.05, **P < 0.01, ***P < 0.01.

Next, we investigated how the gut microbiota composition changes over time in the recipient mice. To this end, the composition of the gut microbiota of the different experimental groups was analyzed with 16S rDNA sequencing. From the GF recipient mice that received old or young microbiota, we analyzed feces 1 week after transfer or 4 weeks after transfer (Figure 5). We were particularly interested to see whether the gut microbiota evolves into a community similar to the donor, or whether it adapts to its host. Redundancy analysis at the genus level confirmed that gut microbiota composition was different between old conventional mice and young conventional mice, since the samples separated into two distinct clusters (Figure 6A). The samples collected 1 week after transfer also separated into two different clusters, suggesting that the transfer of different gut microbiota communities also led to the establishment of different microbiota communities in the recipients. However, after 4 weeks gut microbiota composition in the recipient mice was most similar to the gut microbiota composition in young conventional mice. Moreover, at this time point the clusters of samples derived from the recipient mice were showing more overlap, which suggests gut microbiota composition of the two groups became more similar to each other compared with the first week time point. Together these results indicate that at 1 week the gut microbiota composition of the donor dictates the gut microbiota composition in the recipient, but at later time points the gut microbiota composition adapts to the host.

To look more specifically at the bacterial groups that were responsible for the observed differences in immune responses, we investigated which bacterial phyla had a significant difference in abundance (Figure 6B). Compared with young conventional mice, old conventional mice had higher abundance of *Tenericutes*, but lower abundance of *Verrucomicrobia*. *Akkermansia* is the only genus known to belong to the *Verrocumicrobia* phylum. Indeed we observed a similar difference in abundance for *Akkermansia* (data not shown). In addition, age influenced the *Firmicutes/Bacteroidetes* ratio. Old conventional mice had more *Bacteroidetes*, but less *Firmicutes* compared with young conventional mice. Interestingly, 1 week after transfer of old microbiota, recipient mice had significant less *Verrucomicrobia* than GF mice that received young microbiota (Figure 6B). There was also a difference in the *Firmicutes/Bacteroidetes* ratio, but surprisingly recipients of old microbiota had significantly less *Bacteroidetes* and more *Firmicutes* compared with recipients of young microbiota. Four weeks after transfer, the differences at 1 week were no longer present. However, at this time point recipients of old microbiota had a higher abundance of TM7 and *Proteobacteria* (Figure 6B). The difference in *Proteobacteria* was likely due to a difference in abundance of *Desulfovibrio*, since this was the only *Proteobacterium* that was significantly more abundant at the genus level after transfer of old microbiota. In summary, a number of bacterial groups were identified that were affected by age, which included *Akkermansia*, TM7, and *Proteobacteria*. These bacterial groups are possibly involved in the increased inflammatory potential of the old microbiota.

## Discussion

Several studies have demonstrated that aging is associated with an altered gut microbiota composition, inflammaging, and increased gut permeability (17). However, whether the aged microbiota is a cause or consequence of inflammaging is not known. To the best of our knowledge, we are the first to show that some characteristics of this typical immunosenescence can be induced by microbiota of aged mice after transfer into young GF mice. Microorganisms associated with this effect were found to be *Akkermansia*, TM7 bacteria, and *Proteobacteria*. Our results indicate that increased amounts of TLR2-stimulating components were found in the circulation of recipients of old microbiota, which may have promoted increased inflammation and enhanced T cell differentiation. Interestingly, dysbiosis and a comprised intestinal barrier are also observed in several other disorders such as IBD and metabolic syndrome (23, 24).

Certain bacterial species colonizing the gut have been shown to induce a specific subset of Th cell. For example, segmented filamentous bacterium was found to specifically induce Th17 cells in the gut (25). On the other hand, polysaccharide A produced by the human symbiont *Bacteroides fragilis* was shown to promote expansion of IL-10-producing T_reg_ cells in a TLR2-dependent manner (26–28). It has also been demonstrated that certain *Clostridium* species induce T_reg_ cells in the colon (29, 30). In our study, the aged microbiota did not promote differentiation of a specific Th cell subset. However, after transfer of aged microbiota to GF mice, we rather observed increased levels of several Th cell subsets. This effect was almost exclusively observed in the spleen, but not in the PPs or MLNs. These results do not suggest an association with any of the bacterial species mentioned above. An increased exposure of naïve T cells in the systemic compartment to bacterial compounds in general as a result of a reduced intestinal barrier seems a more likely explanation.

The transfer of old microbiota into young GF mice induced differential regulation of pathways including T cell differentiation, B-cell development, and recognition of microbes by pattern recognition receptors. A central regulatory cytokine was TNF-α, which was consistently upregulated by the old microbiota both in the conventional mice and GF recipient mice. TNF-α is well known for its role in the pro-inflammatory response (31). TNF-α also plays a central role in the pathogenesis of IBD and anti-TNF-α agents are used in the clinic to treat the disease (32). TNF-α was also shown to increase intestinal epithelial permeability (33). Interestingly, in agreement with our data it was recently demonstrated that age-associated inflammation depends on the microbiota and TNF- α (34).

Young microbiota had a different effect and increased expression of lambda immunoglobulin light chain genes both in conventional and GF recipient mice. B cells express only one class of light chain, lambda (λ), or kappa (κ). It has been observed previously that the gut microbiota can influence the ratio of these two light chains. Microbial colonization of GF mice was shown to increase the ratio of Igλ^+^ to Igκ^+^ B cells in the lamina propria (35). Since increased Igλ usage by B cells is considered a marker for B-cell receptor editing (36–38), these results might suggest that the young microbiota promote a more diverse B-cell repertoire. Another possibility is that the young microbiota contains more antigens that are recognized by B-cell clones that express Igλ.

The increased level of differentiated CD4^+^ T cells in the spleen, the elevated inflammation in the ileum, and the prediction of LPS as an upstream regulator in the presence of aged microbiota led us to hypothesize that more bacterial components had translocated into the circulation in animals containing old microbiota. Indeed we observed that serum of GF recipients of old microbiota had an increased ability to activate TLR2 and trend toward increased TLR4 stimulation. Similar mechanisms seem to contribute to other disorders such as type 2 diabetes and metabolic syndrome. High-fat diet was shown to alter gut microbiota composition, which increased the permeability of the small intestine (22). The increased permeability allowed bacterial components to reach distal sites, which induced low-grade inflammation and subsequent insulin resistance (21). Importantly, the mucin-degrading bacterium *A. muciniphila* was shown to reverse these metabolic disorders by strengthening the intestinal barrier (39). In our study, old conventional mice had lower abundance of *Akkermansia*, which has also been reported previously both in humans and mice (40, 41). *Akkermansia* was also less abundant after transfer of old microbiota to GF mice at early time points. Therefore, it is tempting to speculate that the absence of *Akkermansia* in recipients of old microbiota might be associated with translocation of inflammatory bacterial components into the circulation.

As mentioned previously, certain members of the gut microbiota modulate the immune system (42). However, components of the immune system such as IgA antibodies also shape gut microbiota composition (43–45). Therefore, we investigated whether after transfer to GF mice the aged microbiota remained similar in composition to the donor or would quickly adapt to the young host. One week after transfer, the composition of old and young microbiota was clearly different, but after 4 weeks the difference was less pronounced, and both the microbiota from the old and young mice were more similar to the microbiota of the young mice. This suggests that the aged microbiota had partially adapted to the young host. As described for aged humans (8, 9), old conventional mice had a lower *Firmicutes/Bacteroidetes* ratio. However, this trait was not transferable to GF mice. Four weeks after transfer, GF recipients of old microbiota had more TM7 bacteria and *Proteobacteria*. The difference in *Proteobacteria* was at least partially due to a significant lower abundance of *Desulfovibrio* after transfer of old microbiota. Interestingly, *Desulfovibrio* and TM7 bacteria have recently been associated with a compromised intestinal barrier due to an altered mucus structure that was more penetrable by bacteria leading to increased intestinal immune infiltration (46). Further indications that TM7 phyla and *Proteobacteria* such as *Desulfovibrio* can contribute to intestinal inflammation comes from observations that these bacteria are associated with the pathogenesis of IBD (47–49). In conclusion, our data seem to support the hypothesis that the altered gut microbiota composition in aged individuals contributes at least partially to the chronic low-grade inflammatory state observed during aging. Therefore, strategies to modify gut microbiota composition of the elderly with, for example, probiotics or prebiotics (50) might help to reduce inflammation and thereby promote healthy aging.

## Ethics Statement

All animal experiments were approved by the local ethical committee (DEC) of the University of Groningen (project number 6543) and adhered to FELASA guidelines.

## Author Contributions

FF designed the experiments and wrote the manuscript. FF, AB, and TB performed the experiments. SA, FH, and HFS generated and analyzed the microbiota data. CJ and MJ provided material and resources. MB generated and analyzed microarray data. HS, MF, and PV supervised the study.

## Conflict of Interest Statement

The authors declare that the research was conducted in the absence of any commercial or financial relationships that could be construed as a potential conflict of interest.

## Supplementary Material

Figure S1 Gating strategy to identify T helper subsets. Gating strategy and representative FACS plots (spleen sample) for identifying T cell subsets. Among CD3^+^CD4^+^ T cells, the percentage of Th1 cells (T-bet+), Th2 cells (GATA-3+), Th17 cells (RORgT+), and T_regs_ (Foxp3+CD25+) was identified. Percentage of cells stained with isotype control was subtracted to determine the percentage of true positive cells.
