# Supplementary figures and images for: Aged Gut Microbiota Contributes to Systemical Inflammaging after Transfer to Germ-Free Mice
Source: Front Immunol. 2017 Nov 2;8:1385. doi: 10.3389/fimmu.2017.01385 (PMC5674680; doi:10.3389/fimmu.2017.01385)

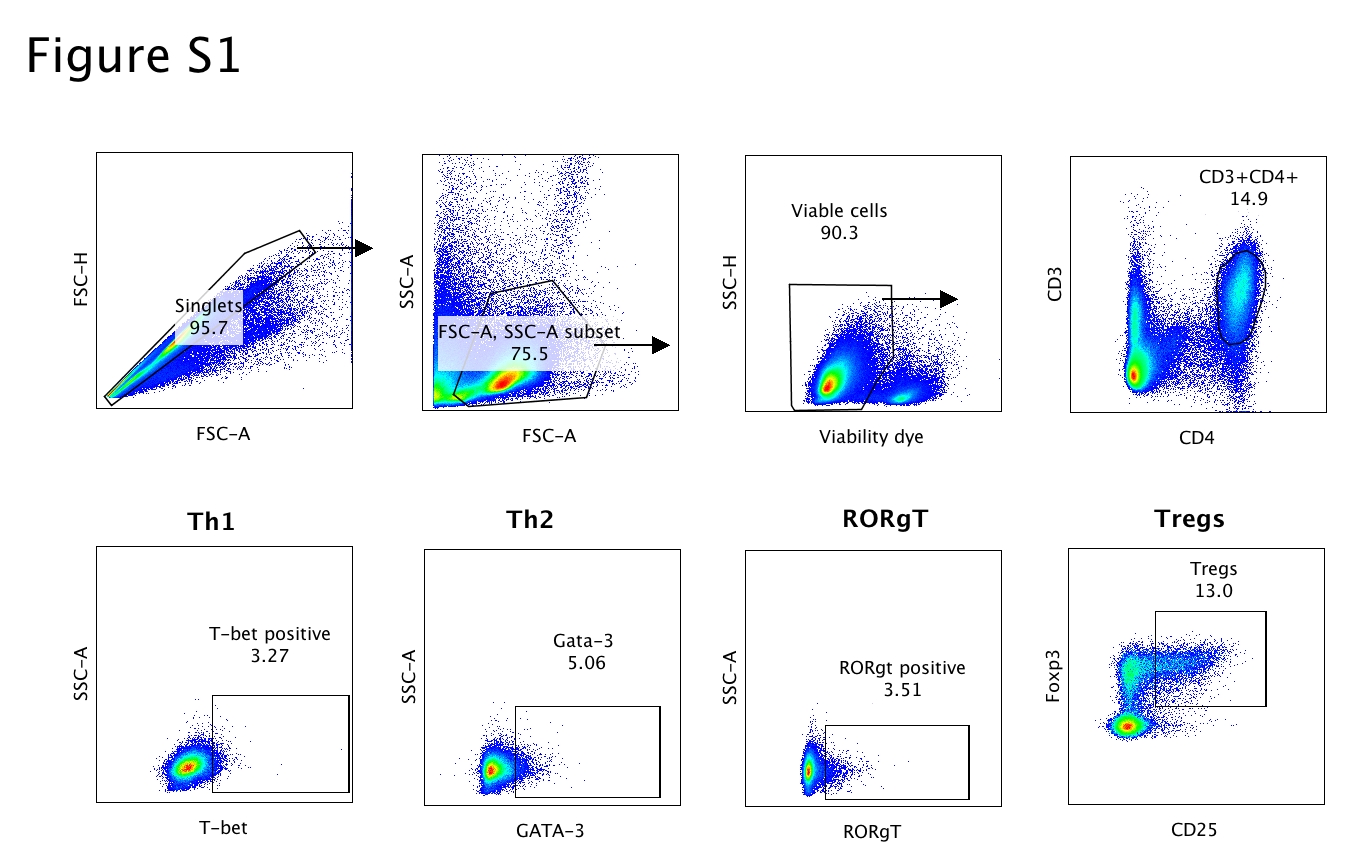

Supplement: Figure S1 — Gating strategy to identify T helper subsets. Gating strategy and representative FACS plots (spleen sample) for identifying T cell subsets. Among CD3+CD4+ T cells, the percentage of Th1 cells (T-bet+), Th2 cells (GATA-3+), Th17 cells (RORgT+), and Tregs (Foxp3+CD25+) was identified. Percentage of cells stained with isotype control was subtracted to determine the percentage of true positive cells. [file Image_1.JPEG]
